# Source Localization of Somatosensory Neural Generators in Adults with Attention-Deficit/Hyperactivity Disorder

**DOI:** 10.3390/brainsci13020370

**Published:** 2023-02-20

**Authors:** Heather S. McCracken, Bernadette A. Murphy, Ushani Ambalavanar, Cheryl M. Glazebrook, Paul C. Yielder

**Affiliations:** 1 Faculty of Health Sciences, Institute of Technology, University of Ontario, Oshawa, ON L1G 0C5, Canada; 2 Faculty of Kinesiology and Recreation Management, University of Manitoba, Winnipeg, MB R3T 2N2, Canada; 3 Health, Leisure and Human Performance Institute, University of Manitoba, Winnipeg, MB R3T 2N2, Canada; 4 School of Medicine, Deakin University, Geelong, VIC 3220, Australia

**Keywords:** Attention-Deficit/Hyperactivity Disorder (ADHD), somatosensory evoked potential (SEP), source localization, sLORETA, motor learning

## Abstract

Attention-Deficit/Hyperactivity Disorder (ADHD) is a neurodevelopmental disorder, where differences are often present relating to the performance of motor skills. Our previous work elucidated unique event-related potential patterns of neural activity in those with ADHD when performing visuomotor and force-matching motor paradigms. The purpose of the current study was to identify whether there were unique neural sources related to somatosensory function and motor performance in those with ADHD. Source localization (sLORETA) software identified areas where neural activity differed between those with ADHD and neurotypical controls when performing a visuomotor tracing task and force-matching task. Median nerve somatosensory evoked potentials (SEPs) were elicited, while whole-head electroencephalography (EEG) was performed. sLORETA localized greater neural activity post-FMT in those with ADHD, when compared with their baseline activity (*p* < 0.05). Specifically, greater activity was exhibited in BA 31, precuneus, parietal lobe (MNI coordinates: X = −5, Y = −75, and Z = 20) at 156 ms post stimulation. No significant differences were found for any other comparisons. Increased activity within BA 31 in those with ADHD at post-FMT measures may reflect increased activation within the default mode network (DMN) or attentional changes, suggesting a unique neural response to the sensory processing of force and proprioceptive afferent input in those with ADHD when performing motor skills. This may have important functional implications for motor tasks dependent on similar proprioceptive afferent input.

## 1. Introduction

### 1.1. Neural Attributes of ADHD

Attention-Deficit/Hyperactivity Disorder (ADHD) is classically defined as a neurodevelopmental disorder, with the most common behavioural characteristics being hyperactivity, impulsivity, and inattention [1,2]. Although ADHD is commonly described as having behavioural characteristics, there are also unique neural attributes associated with ADHD, which are relevant to the current work. For instance, these distinct neural characteristics are present in cortical and subcortical locations, including the prefrontal cortex, anterior cingulate, precuneus, parieto-temporal regions, mesocorticolimbic networks, caudate, thalamus, and cerebellar regions, amongst other locations [3,4,5,6,7,8,9,10,11]. Additionally, predominant neurophysiological characteristics of ADHD are thought to be related to alterations to frontal–striatal–cerebellar network circuity [5,12]. Alterations to fronto-cerebellar circuitry may be strongly related to symptoms of ADHD, including inattention and hyperactivity [13,14]. Cerebellar alterations related to clinical outcomes associated with ADHD have been found to continue through development and into adolescence [15]. Specifically, increased clinical symptoms are associated with reduced volume in inferior-posterior cerebellar lobes [15]. Furthermore, Makris et al. [16] found that adults with ADHD exhibited cerebellar volume reductions. Adults with childhood-onset ADHD exhibit alterations to connectivity within the right hemisphere, particularly within brain regions involved in executive function and attention [9]. This suggests that structural changes associated with ADHD continue into adulthood. Duerden and colleagues noted differences in cortical thickness in sensorimotor processing neural substrates, indicating increased thickness in the pre-SMA and the S1 in those with ADHD [17]. However, the influence that these unique neural characteristics may have on function, particularly those related to somatosensory processing and the acquisition of motor skills in adult ADHD, remains unclear.

### 1.2. Behavioural Characteristics of ADHD

ADHD is common in occurrence, with approximately 11% of children in the United States being diagnosed with ADHD [1]. Although ADHD is commonly described as a disorder predominantly present during childhood, approximately 65% of children diagnosed with ADHD will continue to exhibit symptoms well into adulthood [18]. Currently, limited literature exists addressing the signs and symptoms of ADHD in adulthood, including the neural and behavioural attributes that may be present, and their influence on sensory and motor functions [19,20,21,22,23,24,25]. The hallmark behavioural characteristics described above can have important implications for how individuals function on a day-to-day basis, potentially hindering both mental and physical health in this population [26]. ADHD in adulthood has been associated with increased levels of depression and anxiety, and lower levels of employment, relationship quality, and health and wellbeing, including an increased likelihood of experiencing financial difficulties [27,28,29,30]. This indicates that ADHD in adulthood, although presenting differently from in childhood, may have important implications for both quality of life and functional abilities [27]. Due to the significant effect ADHD symptomology has on daily life, further research is necessary to improve the understanding of unique neural characteristics in ADHD and their potential implications for behaviour. Furthermore, an improved understanding of the neural underpinnings associated with ADHD may aid our comprehension of how alterations to sensory processing may affect day-to-day life in adults.

### 1.3. Somatosensory Function and Sensorimotor Integration

Although there is notably limited literature assessing adult ADHD, the existing literature suggests that somatosensory function and sensorimotor integration (SMI) are altered in this population [17,31,32,33,34,35,36,37,38,39]. Functional and structural alterations to neural substrates involved in sensory integration may be related to performance-based outcomes. For instance, ADHD is associated with difficulties performing tasks that require motor coordination and performance [40,41,42]. A potential hallmark of ADHD symptomology is deficient inhibitory motor control [43]. Additionally, difficulties exist in performing tasks dependent on motor coordination, including balance during a single task, walking, reaction time, motor timing, slower movement preparation, and handwriting [40,44,45,46,47,48]. Difficulties in motor acquisition and performance associated with ADHD are likely related to, at least in part, alterations in sensorimotor processing.

Our previous study established the presence of an increased N18 and reduced N30 somatosensory evoked potential (SEP) peak when utilizing a visuomotor tracing (MTT) paradigm in adults with ADHD when compared with neurotypical controls [20]. Furthermore, a reduction in the N18 peak in those with ADHD after acquiring a novel motor task dependent on force modulation (FMT) was present [21]. A reduction in the N18 after the FMT likely reflects reduced inhibitory activity of networks relating to olivary–cerebellar–M1 connectivity [49,50,51,52,53], where a reduction in the N18 may be reflective of a filtering effect for continual refinement of motor output [50]. This may be indicative of difficulty processing the force and proprioceptive sensory afferents that are paramount to the performance of the FMT. Results from our previous study, which utilized standardized low-resolution brain electromagnetic tomography (sLORETA), suggest that those with ADHD have attenuated activity within the right-hemispheric Brodmann area (BA) 2 when presented with an audiovisual multisensory stimulus [54]. Studies have noted a role of the right-hemispheric parietal lobe with spatial attention processes [55,56,57,58,59], whereas BA 2, in particular, is reflective of neural processing for complex touch, joint position sense, and pressure [60]. These novel findings provide insight into the role of specific neural regions in the process of motor acquisition and somatosensory processing related to ADHD in adulthood. Furthermore, they suggest the importance of applying sLORETA and SEP techniques to provide an improved understanding of the neural characteristics associated with ADHD. Although pairing surface EEG and SEP techniques was an important first step in assessing somatosensory processing associated with ADHD, and has provided invaluable insight into the processes related to SMI and motor learning, the analysis techniques utilized within previous studies were restricted to surface assessments of cortical activity [20,21], thus limiting the spatial acuity of the assessment. Given the fundamental importance of motor learning and performance in relation to daily function, utilizing neurophysiological techniques which allow for an assessment of neural source location is an important next step which we aimed to address in the current study.

### 1.4. Source Localization

Source localization is a neural technique that pairs collected EEG datasets with a standardized magnetic resonance imaging (MRI) head model. This makes it possible to localize specific neural generators with high spatial acuity. sLORETA offers a non-invasive and cost-efficient technique to analyze neural activity within neural generators [61]. sLORETA has improved spatial resolution of neural structures when compared with an analysis strictly using surface-electrode EEG. The brain map that sLORETA utilizes is the Montreal Neurological Institute (MNI) MRI brain map (MRI-152) [61]. From there, sLORETA performs a linear inverse algorithm, providing an estimated 3D distribution of the sources of neural generators within the human cortex. Furthermore, sLORETA provides a low localization error when being compared with similar techniques that also use a linear inverse algorithm [61]. One functionality of this software is that it is capable of source-localizing EEG data in the time-domain.

### 1.5. Rationale and Purpose

The rationale and purpose for the current study was to further improve our understanding of the neural characteristics and substrates in those with ADHD, particularly those related to motor learning and sensorimotor processing. This can be achieved by applying a form of neural assessment with a high level of spatial acuity, such as sLORETA. The research question addressed within the current study, is: Are there differences in neural activity source locations during visuomotor and/or force modulation tasks in young adults aged 18–35 years old with ADHD? The current study aimed to answer this question for two motor acquisition paradigms, one that is highly dependent on visuomotor processing, and the other that is more so dependent on force modulation and proprioception, thus allowing for an assessment of neural sources involved in motor paradigms that utilize differing sensory pathways. We hypothesize that adults with ADHD will exhibit differences in the source of neural activity after learning novel motor paradigms when compared with neurotypical controls. Specifically, differences may be present within networks heavily involved in somatosensory processing and SMI, such as the primary somatosensory cortex (S1) within parietal regions and those related to the cerebellum due to their involvement in motor performance.

## 2. Materials and Methods

### 2.1. Ethical Approval

This study received ethical approval from the Ontario Tech University Research Ethics Board (REB; # 15307). All participants gave written informed consent before they participated in this study. This study was performed according to the principles set out by the Declaration of Helsinki for the use of humans in experimental research.

### 2.2. Participants

Participants were students from the Ontario Tech University campus. All participants were between 18–35 years of age. Two motor paradigms were assessed in this study, and each paradigm had one group of young adults with ADHD and one group of neurotypical controls. Participants were the same as those included in our previous studies looking at cortical activity using evoked potentials [20,21]. Those in the ADHD group had a previous clinical diagnosis of ADHD from their health care provider. Two paradigms were used in this study: a novel visuomotor tracing task (MTT) and a novel force-matching task (FMT). Those with ADHD (*n* = 15; 9 females) in the FMT group had a mean age of 22.00 ± 2.51, while controls (*n* = 15; 9 females) had an average age of 20.80 ± 1.97. Participants in the ADHD group (*n* = 12; 8 females) for the MTT had a mean age of 21.5 ± 1.93, while controls (*n* = 16; 9 females) had a mean age of 20.81 ± 2.46.

All participants reported that they were right-handed, and their handedness was further confirmed using the Edinburgh Handedness Inventory (EHI). Additionally, participants completed questionnaires as part of pre-screening before participating, to ensure they did not have a history within the past five years of concussion, brain injury, epilepsy, or stroke that could have inadvertently affected the EEG, SEPs, and sLORETA results. All participants completed the Adult ADHD Self-Report scale (AASRS-v1.1) pre-screening questionnaire prior to participation. The AASRS-v1.1 quantifies symptomology related to ADHD. The AASRS is made up of 18 questions that are strongly correlated to ADHD diagnostic criteria set out by the DSM-IV [62]. All questions are scored on a five-point Likert scale, and scores range from “never” to “very often”. This is an effective tool for predicting ADHD symptomology [63]. The checklist is broken up into part A and part B. Part A is related to inattentiveness, whereas part B is related to hyperactivity and impulsivity. To be clear, no specific score indicates a diagnosis of ADHD; rather, it enables a quantification of symptoms associated with ADHD, and therefore to compare between groups (ADHD vs. control). Those in the ADHD group who completed the FMT task had an average score of 22.40 ± 4.44 for part A (Controls: 14.27 ± 4.46) and 44.07 ± 8.16 (Controls: 24.93 ± 6.18) for part B. For the MTT, those in the ADHD group had an average score of 21.58 ± 4.71 for part A compared with Controls, with an average score of 12.31 ± 3.53, and a mean score for part B of 42.33 ± 8.03 (Controls: 22.94 ± 5.73).

### 2.3. Procedures

#### 2.3.1. SEPs Stimulation Parameters

The SEPs stimulation frequency was set at 2.47 Hz. SEPs were delivered via stimulation of the median nerve just proximal to the right wrist, which was approximately 2 cm proximal to the distal crease of the wrist. The stimulation intensity was set to the motor threshold of the abductor pollicis brevis (APB), being the lowest intensity at which a visible thumb twitch of 1 cm in amplitude occurred. The noted muscle contraction occurs due to stimulation of the median nerve, which is a mixed nerve. This will ensure that the 1a afferents are stimulated, which is a fundamental part of eliciting the short-latency SEP peaks, as a result of their cortical projections [64]. The anode of the stimulating electrodes is placed proximal, and the cathode is distal in relation to the wrist. Stimuli are sent via a Digitimer Stimulator (Digitimer DS7A constant current, Welwyn Garden City, UK). These stimulations were delivered as square pulses, 200 µs in duration, at a constant frequency via Ag/AgCl EMG conductive surface electrodes (Meditrace™ 130, Kendall, Mansfield, MA, USA). Stimulations were delivered for 1000 sweeps, allowing for clearer averages of individual SEP peaks. Stimulations occurred prior to and immediately after each of the novel motor learning paradigms occurred (MTT and FMT).

Signal4 software (Version 4.08, Cambridge Electronic Design, Cambridge, UK) was used to record the peripheral SEP peaks, including the N9 which was recorded over the ipsilateral brachial plexus, or Erb’s point [52]. The N9 was referenced to the ipsilateral earlobe using electrode paste and an ear clip electrode [52]. Based on the IFCN guidelines, the N9 SEP peak must remain stable (±20%) from pre- to post-measures for each participant in order for their data to be included in the rest of the analyses [65].

#### 2.3.2. EEG Collection Parameters

Cortical electrical activity was recorded using surface EEG in the form of a Waveguard™ 64-electrode EEG cap (ANT Neuro, Hengelo, The Netherlands). The Waveguard™ cap was connected to the TMSi REFA-8 amplifier (TMSi, Oldenzaal, The Netherlands) with 64 EEG channels, 4 bipolar channels, and 4 auxiliary channels. EEG data were collected using Advanced Source Analysis Lab™ (ANT Neuro) software, and all EEG signals were collected at a 2048 Hz sampling frequency. Each electrode had an impedance below 10 kΩ.

#### 2.3.3. Paradigm(s)

##### Novel Visuomotor Tracing Task (MTT) Parameters

**Figure 1:**
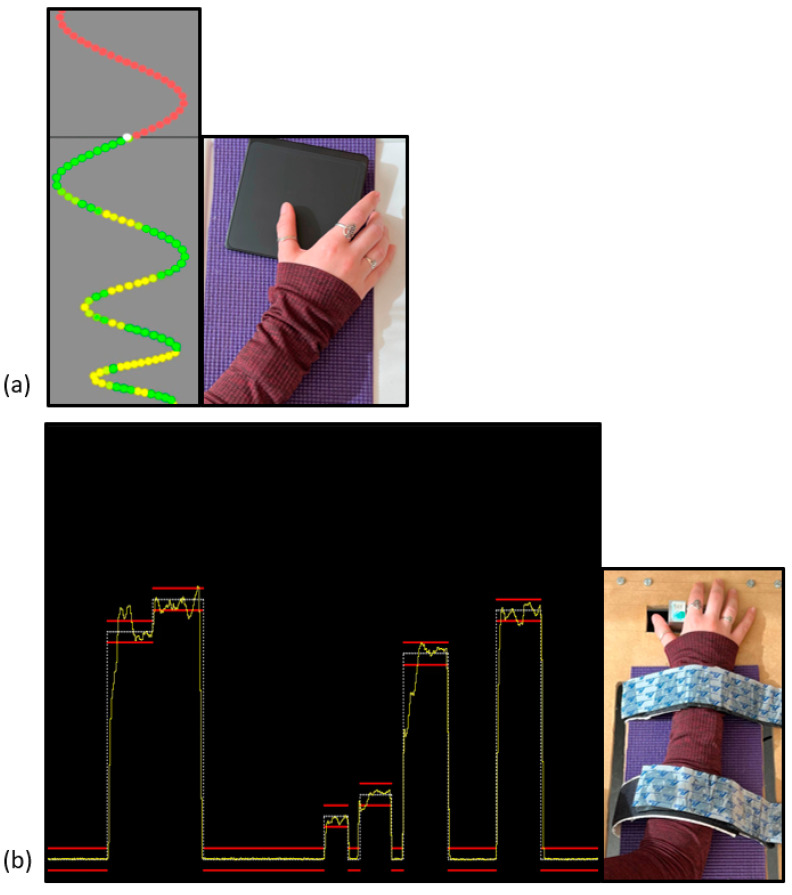
Depiction of each motor paradigm. (**a**) Visuomotor tracing task (MTT) and (**b**) force-matching task (FMT). Each task was completed using the right hand and thumb, while visual feedback was presented to the participant.

The novel MTT was delivered via a custom Leap Motion software tool (Leap Motion, Inc., San Francisco, CA, USA), which was launched using Unity™ software developed for gaming. This paradigm consisted of sinusoidal waveform patterns, with four unique traces that varied in intensity and frequency, thus allowing for variability in how difficult each trace was. This variation allows for an unpredictable task, potentially enabling learning to occur [66]. Traces were presented in a pseudo-randomized order, to avoid any order effects, potentially affecting learning processes [67]. The waveforms were a continuous stream of coloured dots that moved vertically down the computer monitor. The MTT paradigm can be seen in Figure 1a. A red dot indicated that that particular dot had not been traced yet; green indicated a perfect match; and variations of yellow-green indicated an imperfect match. To trace this waveform, thumb abduction and adduction were necessary, and were performed on a wireless mouse touchpad (Logitech, Inc., Fremont, CA, USA). This task was completed in phases of 4 pre-acquisition blocks, 12 acquisition blocks, and 4 post-acquisition blocks, similar to that of the FMT behavioural paradigm. SEPs were collected prior to and immediately after participants completed this novel motor paradigm.

##### Novel Force-Matching Task (FMT) Parameters

The novel FMT was delivered in several blocks, including 4 pre-acquisition blocks, 12 acquisition blocks, and 4 post-acquisition blocks. A block is defined as a group of trials, and each trial consisted of 3–5 traces. This task required force-modulation of the right thumb via adduction and abduction, enabling participants to trace a waveform on the screen. This can be seen in Figure 1b. The waveform varied in force, which was based on the maximal voluntary contraction (MVC) that was collected prior to the start of the motor paradigm. The MVC was an average of three trials, and was based on the strength of their APB muscle. The traces were delivered via a computer monitor that was placed directly in front of the participant, and the force-transducer was attached to a height-adjustable table.

The task was delivered using LabVIEW custom programming (National Instruments, Austin, TX, USA). The force transducer utilized was calibrated using a 50 kg load cell. Each trace varied from 2% to 12% of each individual’s APB MVC. The intended trace was a series of white dots, and participants saw their force output on the computer monitor via a yellow line. Two horizontal red bars were placed on each side of the trace (white line), and these were placed 0.5% ± the white line, acting as a guide or boundary within which participants aimed to stay. While completing the behavioural paradigm, the participant’s hand was pronated with their thumb resting against the transducer. SEPs were collected immediately prior to and after completion of this novel FMT paradigm.

### 2.4. Data Processing and Statistical Analysis

#### 2.4.1. EEG/SEPs

EEG data processing occurred offline, using ANT 4.10.1 software, in order to remove artifacts, such as those from blinking, from the EEG signal. A band-pass filter with a low cut-off of 0.2 Hz and a high cut-off of 1000 Hz, and a slope of 24 dB/octave, was used. This process was performed for all datasets. All EEG datasets were then averaged and epochs were created starting from −10 ms and to 200 ms, making for a total epoch duration of 210 ms. This allowed for the assessment of all short-latency SEP peaks. Each participant’s data had two averages, one from the “pre” or baseline stimulation prior to the motor paradigm, and one from the “post” stimulation measures that occurred directly after completion of the motor paradigm.

#### 2.4.2. Source Localization–sLORETA Analysis

sLORETA software was used to perform the source localization analyses [61,68,69]. sLORETA software is a linear inverse algorithm and works as a method to solve the inverse problem, based upon an assumption that neighbouring neurons activate in a synchronous and simultaneous manner, and this is completed without a localization bias [61,70,71]. This program has been validated for its accuracy, and was carried out using both EEG and fMRI data [72], indicating that the estimated sources of neural activity found using sLORETA are reliable. The sLORETA template divides cortical grey matter into 6239 voxels, with a 5 mm spatial resolution. In total, 5000 permutations based on statistical nonparametric mapping (SnPM) were performed on the voxel-wise randomization tests. This process of randomization corrects for multiple comparisons, providing the greatest statistical power possible [73]. The MNI average MRI brain-map (MNI-152), including the associated head model and electrode coordinates, were used to calculate the standardized current density at each voxel. sLORETA analysis can compare sources of neural activity between groups.

This analysis was performed in the time-domain, for the following comparisons:

(1) Between groups (ADHD vs. control) at both baseline and post measures. Comparisons were performed for both the MTT and FMT.
  - Baseline ADHD vs. baseline control, to assess potential group differences at baseline measures.
  - Post ADHD vs. post control, assessing group differences in source activity after the acquisition of the motor paradigm.
(2) Within groups (pre-measures vs. post-measures) for both the ADHD and control group. Similarly, comparisons were performed for both tasks, the MTT and FMT. This comparison was performed to assess whether locations of source activity differed within each group after acquisition of either of the motor paradigms.
  - ADHD baseline vs. ADHD post.
  - Control baseline vs. control post.
(3) Finally, comparing between tasks, to assess whether somatosensory neural processing differed significantly between the two task conditions (MTT vs. FMT), discerning neural sources were activated in response to visuomotor vs. force-matching demands of each task, respectively.
  - ADHD
    - Baseline MTT vs. baseline FMT.
    - Post MTT vs. post FMT.
  - Control
    - Baseline MTT vs. baseline FMT.
    - Post MTT vs. post FMT.

#### 2.4.3. Statistical Analysis in the Time-Domain

All tests had statistical significance set at *p* = 0.05. All statistical tests were performed within sLORETA’s statistical tool in the time-domain [61,74]. Statistical tests in sLORETA were performed using an independent (between group) and paired (within group) two-tailed Student’s *t*-test, depending on the comparison being performed. First, this converts all of the EEG data values into t-values for each of the time frames. This process was completed on 430 time frames, as the epoch was 210 ms in duration and was collected at a 2048 Hz sampling frequency. The software provides a t-critical value in the form of a two-tailed t-value threshold. The SnPM adjusted for multiple comparisons by utilizing 5000 randomized permutations [73]. Once the t-critical threshold is established, the t-value output is assessed, and if a t-value exceeds t-critical, sLORETA software will then perform a computation that localizes the neural location where the difference in activity occurred. In conjunction with this, the software provides the statistical significance (*p*-value) associated with the difference. Thus, this illustrated whether the differences noted were statistically significant or not. Furthermore, an ASA Lab™ (ANT Neuro) sLORETA source localization tool was utilized to generate individual graphical images of neural activity for all groups, where sLORETA had established a significant change during the time-domain analysis described above. This was performed on the latency at which the sLORETA analysis had elucidated the presence of neural areas where there was significantly different activation between tested groups, and produced an enhanced visual representation of activation sources for each group separately.

## 3. Results

**Figure 2:**
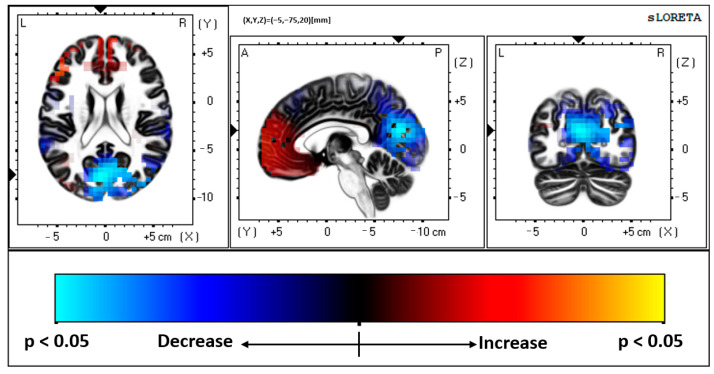
sLORETA image, depicting area of neural activity where the greatest difference occurred between pre and post conditions in those with ADHD in the FMT condition (*p* < 0.05).

**Figure 3:**
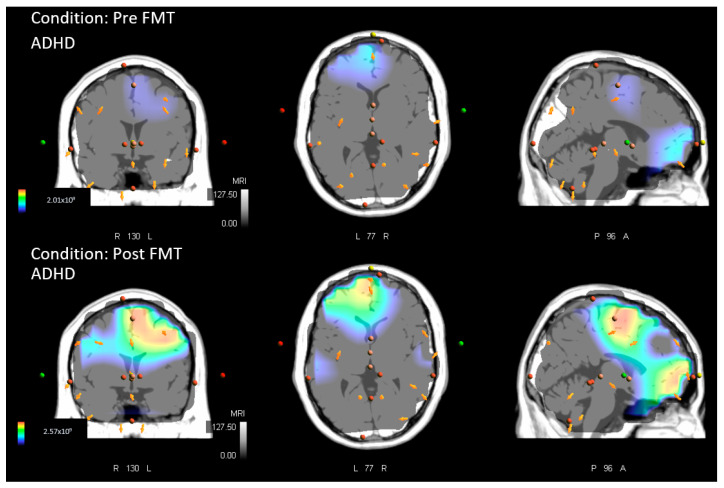
Sources of neural activity in those with ADHD at baseline (pre) measures and at post-measures after participants completed the FMT. Latency coincides with the differential image created using sLORETA (Figure 1). Cross-sectional areas include the coronal (**left**), transverse (**middle**), and sagittal plane (**right**).

**Figure A1:**
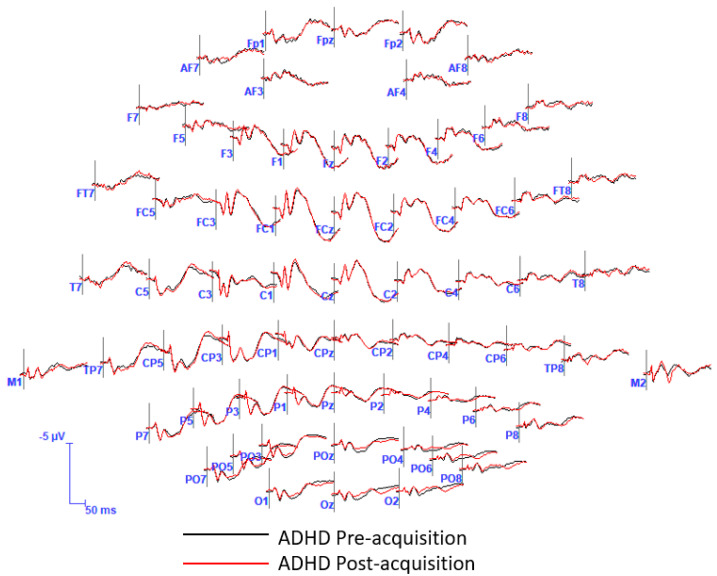
Depiction of the FMT pre (baseline) and post grand-average ERP waveforms for the ADHD group. This illustrates the distribution of scalp activity from all electrodes at the latency of—10 ms to 200 ms after median nerve stimulation.

**Figure A2:**
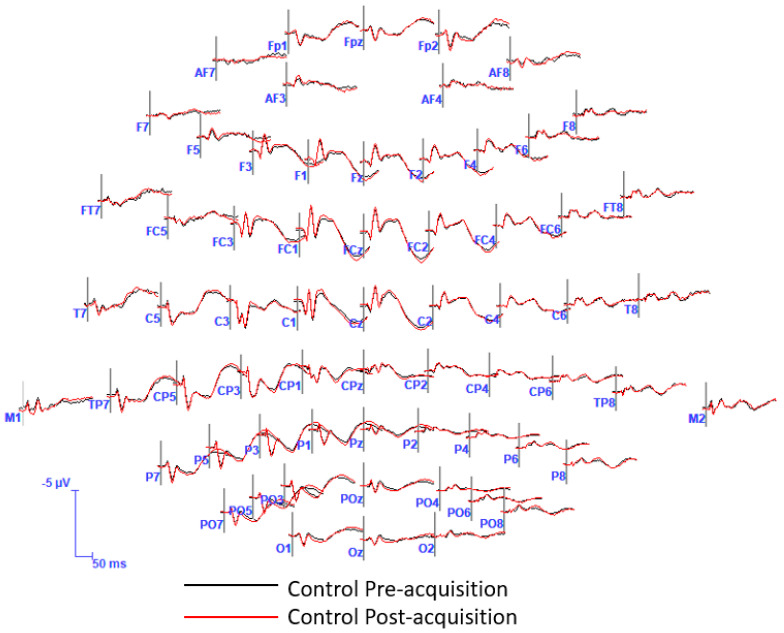
Depiction of the FMT pre (baseline) and post grand-average ERP waveforms for those in the Control group. This illustrates the scalp activity within all electrodes from a latency period of—10 ms to 200 ms after median nerve stimulation.

**Figure A3:**
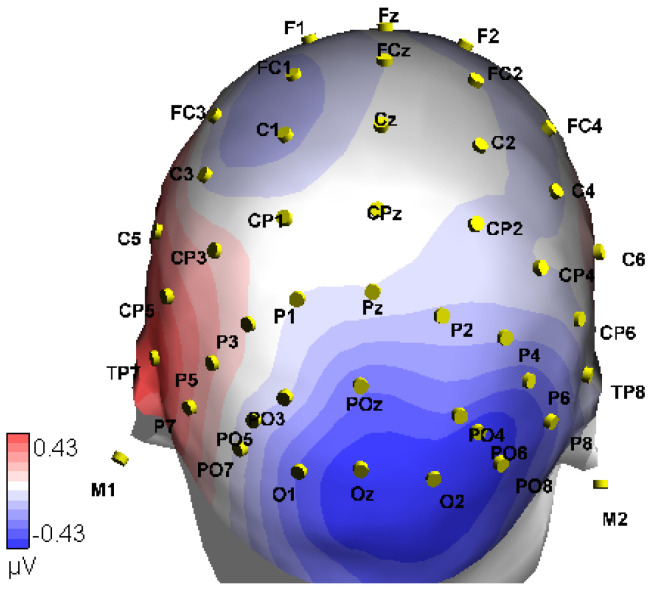
Scalp distribution of the FMT pre-acquisition and post-acquisition differential activity (pre–post) for those in the ADHD group. Latency coincides with that of the sLORETA results in the manuscript.

ADHD Pre vs. ADHD Post, FMT Analysis: the results indicated that those with ADHD had increased neural activity at post-SEP measures (or reduced at baseline/pre), after performing the novel FMT motor paradigm, when compared with their baseline SEP measures. Significantly greater activity was present in BA 31, precuneus, parietal lobe (MNI coordinates: X = −5, Y = −75, Z = 20; *p* < 0.05). Increased activity at BA 31 occurred at approximately 156 ms post median nerve stimulation. Figure 2 depicts the activity difference localized to BA 31 between pre and post conditions in those with ADHD. Figure 3 demonstrates the pre and post FMT ADHD activity separately. Additionally, figures of the grand average SEP waveforms and EEG scalp distribution related to the FMT can be found in the Appendix A Figure A1, Figure A2 and Figure A3.

**Figure 4:**
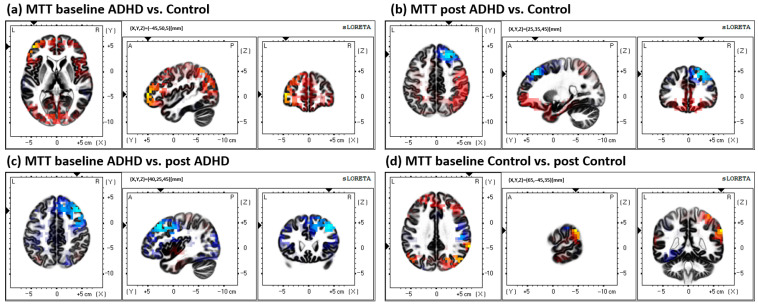
All MTT comparisons. All results yielded were insignificant (*p* > 0.05). (**a**) Comparison of baseline activity between groups (ADHD vs. Control; control activity > ADHD activity), differential activity in BA 10, middle frontal gyrus, frontal lobe. (**b**) Comparison of post-acquisition activity between groups (ADHD vs. Control; control activity < ADHD activity); the greatest difference was found in BA 8, middle frontal gyrus, frontal lobe. (**c**) Within-group ADHD comparison of baseline activity to post-acquisition (baseline < post activity), differential activity found in BA 8, middle frontal gyrus, frontal lobe. (**d**) Within-group control comparison between baseline and post-acquisition (baseline > post), greatest activity difference in BA 40, supramarginal gyrus, parietal lobe.

**Figure 5:**
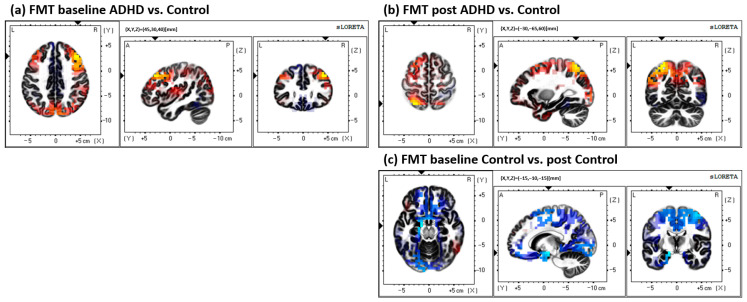
All FMT comparisons. All results yielded were insignificant (*p* > 0.05). (**a**) Comparison of baseline activity between groups (ADHD vs. Control; control activity > ADHD activity), differential activity in BA 9, middle frontal gyrus, frontal lobe. (**b**) Comparison of post-acquisition activity between groups (ADHD vs. Control; control activity > ADHD activity); the greatest difference was found in BA 7, superior parietal lobule, parietal lobe. (**c**) Within-group control comparison between baseline and post-acquisition (baseline activity < post activity), greatest activity difference in BA 28, parahippocampal gyrus, and the limbic lobe.

**Figure 6:**
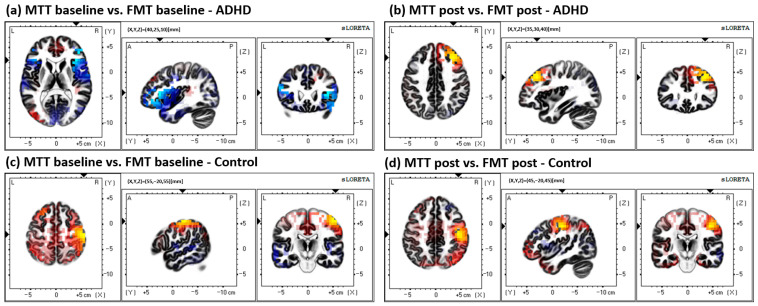
All between-task (MTT vs. FMT) comparisons. All results yielded were insignificant (*p* > 0.05). (**a**) Comparison of baseline activity between tasks in the ADHD group (MTT < FMT), differential activity in BA 13, inferior frontal gyrus, frontal lobe. (**b**) Comparison of post-acquisition activity between tasks in the ADHD group (MTT activity > FMT activity); the greatest difference was found in BA 9, middle frontal gyrus, and the frontal lobe. (**c**) Between-task baseline activity comparisons in the control group (MTT > FMT); the greatest difference was found in BA 1, postcentral gyrus, and the parietal lobe. (**d**) Between-task post-acquisition activity comparison in the control group (MTT > FMT); the greatest activity difference was found in BA 4, precentral gyrus, frontal lobe.

All other comparisons are outlined in the methods: all other statistical tests, including within and between groups for both the MTT and FMT, yielded non-significant differences (*p* > 0.05). Results from the MTT comparisons can be seen in Figure 4; results from the FMT comparisons can be seen in Figure 5; and results from the between-task comparisons (MTT vs. FMT) can be seen in Figure 6. All results depicted in Figure 4, Figure 5 and Figure 6 are not statistically significant (*p* > 0.05).

## 4. Discussion

To the best of our knowledge this is the first study to assess the source location of neural generators pertaining to somatosensory processing and motor learning in young adults with ADHD. The novel results from the current study indicate that when performing a motor paradigm dependent on force modulation and proprioception, those with ADHD have a greater activation in BA 31, precuneus, and the parietal lobe, at post measures after acquiring this novel motor skill. This difference in neural activation was present at a latency of 156 ms post median nerve stimulation. When taking into consideration both the region of neural activation as well as the latency, this provides invaluable insight into neural processing in those with ADHD in response to completing a motor paradigm that is highly contingent on proprioceptive feedback for success. This is of particular relevance to many day-to-day tasks which often require the neural processing of proprioceptive afferents. For instance, many motor skills require force modulation, such as when applying pressure to a pedal in a car, interacting with various remotes such as those in virtual reality settings, and for many occupational skills, such as surgeons who use forceps. We did not find differences in source activity with any of the other comparisons. This suggests that the source of neural activity remained relatively consistent in neurotypical controls for both motor paradigms and during the visuomotor tracing task in those with ADHD. The lack of differences in the sources of neural activity in the other comparisons may be a result of similar neural sources being present between all compared groups, which may have been in contrast to the profound differences present in those with ADHD when completing the FMT at baseline when compared with post-acquisition. The difference found reflects that of unique processing in adults with ADHD after performing a motor task that requires the utilization of force-modulation. Postulated mechanisms for such differences after acquiring the novel FMT in those with ADHD, including potential explanations, will be discussed further below.

### 4.1. Brodmann Area (BA) 31

BA 31, which is also commonly referred to as dorsal posterior cingulate area 31, is located at the medial border of the parietal lobe, between the splenial sulci and the cingulate, and includes cortices of the precuneate and the posterior cingulate [75,76]. The localized neural generator within the current study was specifically BA 31 and the precuneus. One of the key roles of the posterior cingulate cortex is in relation to the default mode network (DMN) [77]. The DMN describes a neural network including a number of brain regions, which exhibit deactivation or a reduction in activity during cognitively demanding tasks [78]. Broadly speaking, the DMN encompasses several brain regions, including the lateral temporal cortex, dorsal medial prefrontal cortex, posterior cingulate, ventral medial prefrontal cortex, inferior parietal lobe, and the hippocampus [79]. Previous studies have suggested the presence of altered connectivity between cortical regions, including the precuneus and the anterior cingulate and DMN regions, such as the ventromedial prefrontal cortex, in adult ADHD [6]. The reduction in neural activation in the DMN during attentionally demanding cognitive tasks is explained as this region being reflective of memory recollection or daydreaming [79]. However, there are competing hypotheses for the role of the DMN during cognitive tasks, one of which suggests an active role in working memory [80]. When an individual performs a task that requires focused attention, such as goal-directed behavioural tasks, generally, there will be an attenuation of activity within the posterior cingulate cortex, which is reflective of a reduction in resources being allocated to this neural area during such tasks [78]. This is termed ‘task-induced deactivation’, which is most prominent along the midline of the brain [79]. The activity difference in the current study was specific to the posterior cingulate cortex (BA 31) and precuneus. The current study elucidated the presence of increased neural activation in those with ADHD within cortical structures underpinning this network, after completing the novel FMT motor paradigm. Interestingly, the DMN is altered in those with autism spectrum disorder (ASD), where they fail to exhibit this deactivation [81]. Another hypothesis for the role of the DMN during cognitive tasks is explained as an exploratory state, when an individual has low levels of attention dedicated to monitoring the external environment for unexpected events in an unfocused manner, as a form of information gathering [79,82,83]. This is in contrast to a task that requires a high level of attention on a specific target, such as visual acuity to a stimulus.

The increased activity at BA 31, parietal lobe, in those with ADHD after performing the novel FMT provides important insight into neural function in response to this task. The DMN is commonly described as being altered in those with ADHD [6,11,84,85,86], and additionally altered precuneus connectivity within the DMN is associated with ADHD [87]. ADHD is correlated with connectivity alterations to the DMN, and this is likely due to alterations within frontal–striatal–cerebellar networks [85]. The increased activity within the posterior cingulate cortex and precuneus in the current study may reflect a reduction in attention after the motor acquisition paradigm. In other words, it is possible that those with ADHD experienced difficulty focusing while performing the FMT, resulting in an attenuation of attentional resources at post measures. This may be related to the attenuated activity within BA 2, the right-hemispheric parietal lobe noted in our previous study [54]. BA 2 has a primary role in the processing of pressure, joint position sense, and complex touch [60], thus informing proprioception. Furthermore, the right-hemispheric parietal lobe reflects neural processing associated with spatial attention [55,56,57,58,59]. Therefore, the results from our previous study [54], potentially reflective of attenuated neural processing of proprioceptive and spatial attention, may be related to the increased activity within the DMN of the current study. If this is the case, there are a number of potential reasons why this may have occurred in this particular group. One explanation for this increased activity in the DMN, which is the opposite of what is generally expected during a goal-directed movement, where typically there would be an expected task-related deactivation, may be reflective of those in the ADHD group experiencing difficulty maintaining focus on the task. Inattention to the task at hand may be a result of the task being deemed “too boring” for this particular population, or possibly that it was difficult for them due to noted sensory processing impairments related to force matching and proprioception, thus resulting in a general disinterest for them. Of potential relevance to this is that upon session completion, some participants in the ADHD group stated that they found the novel FMT to be “boring”. However, this is anecdotal, and in the future, incorporating a qualitative measure of self-perceived attention or engagement may aid in elucidating this potential relationship. Another interesting, yet important, variable in the current finding is the latency at which this difference occurred, as it aligns with a mid–late-latency SEP peak, as opposed to short-latency SEPs, which were the objective of the previous studies [20,21].

### 4.2. Latency

The latency at which the difference in activation was present in BA 31 is in line with mid–late-latency SEP peaks, as opposed to that of short-latency SEP peaks. Specifically, the latency of 156 ms may align with the somatosensory N140 peak. The N140 is often observable between 150 and 210 ms [88]. The N140 SEP peak is typically recorded over central or parietal brain regions, with the greatest amplitude over the midline or vertex electrodes, and activity is correlated with selective attention [89,90]. The N140 peak is commonly observable after median nerve stimulation [90]. There is limited information specifying the neural generators underlying this activity [90,91]. However, the primary and secondary sensory areas, the prefrontal area, and the supplemental motor area are all cortical regions thought to be involved in the N140 [91]. Spatial attentional modulation affects the amplitude of the N140 [92,93]. Additionally, the N140 is thought to reflect the processing of tactile information [88], and is also related to cognitive functions, such as those related to selective attention and conscious stimulus perception [94,95,96]. Those with adult ADHD exhibit reductions in right-hemispheric superior longitudinal fascicle II (SLF II) connectivity, and the SLF II is related to visual spatial attention, providing input to the prefrontal cortex from parietal regions [9]. Alterations to the N140 in those with autism are thought to be related to excitation–inhibition balance and circuit hyperexcitability [88]. Therefore, the results from the current study suggesting increased activity post FMT in those with ADHD may be a result of more resources being allocated for spatial attention or awareness. It is postulated that the N140 is related to motor execution and neural activity related to inhibition processing [97,98,99,100,101]. This can be seen as an increased amplitude response during NoGo trials and diminished in response to Go trials [101]. Although not directly related to somatosensory input, previous work utilizing visual afferents noted that the visual N140 was increased in those with ADHD [102]. This increased activity was localized to BA 30, right posterior cingulate, in adults with ADHD, and is potentially due to increased attentional and cognitive demands [102]. Therefore, the activation within neural networks surrounding the latency of the N140 may pose an important area for future research to better elucidate sensory processing and neural function in those with ADHD.

The increased activity within BA 31 at a latency that coincides with the somatosensory N140 after completing a novel motor paradigm that requires force-modulation within the current study may suggest greater cognitive demands allocated to focus on body schema and proprioception associated with the limb and digit completing the task (i.e., right thumb) in those with ADHD. Alternatively, this may relate to self-perceived difficulties with the task, because if participants found the FMT difficult or boring, they may have experienced difficulty maintaining focus during the motor acquisition paradigm. One way to account for this in the future may be to ask participants to rate their mental state/attention before, during, and after performing a task. This would allow for a qualitative assessment of attentional levels at the different stages of the task, and then could aid in the interpretation of the neurophysiological results.

### 4.3. Limitations

Potential limitations include the participants being limited to young university-aged adults with ADHD; therefore, it is unknown whether these results can be generalized to ADHD in childhood or older adulthood. Additionally, although sLORETA in conjunction with high-density EEG is a valid and cost-efficient form of neural assessment, in the future, incorporating neurological techniques, such as functional MRI (fMRI), for each participant would further enhance these findings. The sample sizes for both the FMT and MTT protocols were modest, and although all samples met the sample size goal defined upon study inception, future work may benefit from increased sample sizes in order to ensure that possible additional differences are not missed due to a type II error.

## 5. Conclusions

This study demonstrated that young adults with ADHD exhibit increased activation within BA 31 after performing a motor learning paradigm dependent on force modulation; this increased activity was localized to the precuneus, parietal lobe. The increased activity in BA 31 may reflect up-regulation in the DMN at post measures, in addition to alterations to selective spatial attention after such motor tasks. These findings are specific to motor tasks dependent on force, and were absent when assessing changes after the visuomotor task. Furthermore, these findings are in line with the neurophysiological characteristics associated with ADHD, such as unique functioning of the DMN and precuneus, while adding important contextual insight into the role that these networks play in motor acquisition and learning in adult ADHD. Overall, greater neural activity has a focal point at BA 31 after force-modulation motor tasks in young adults with ADHD, and this provides further insight into the neural functioning relevant to daily motor skills that are heavily dependent on this form of sensory processing.

## Data Availability

The data presented in this study are available in Figure 2 and Figure 6; spreadsheets can be made available upon request.
